# First identification of mammalian orthoreovirus type 3 in diarrheic pigs in Europe

**DOI:** 10.1186/s12985-016-0593-4

**Published:** 2016-08-12

**Authors:** Davide Lelli, Maria Serena Beato, Lara Cavicchio, Antonio Lavazza, Chiara Chiapponi, Stefania Leopardi, Laura Baioni, Paola De Benedictis, Ana Moreno

**Affiliations:** 1 Istituto Zooprofilattico Sperimentale della Lombardia e dell’Emilia Romagna, IZSLER, Via Bianchi 9, 25124 Brescia, Italy; 2 Istituto Zooprofilattico Sperimentale delle Venezie, IZSVE, Viale dell’Università 10, Legnaro Padova, 35020 Italy

**Keywords:** Orthoreovirus, PED, Swine, Bat, Human, Phylogenetic analysis

## Abstract

Mammalian Orthoreoviruses 3 (MRV3) have been described in diarrheic pigs from USA and Asia. We firstly detected MRV3 in Europe (Italy) in piglets showing severe diarrhea associated with Porcine Epidemic Diarrhea. The virus was phylogenetically related to European reoviruses of human and bat origin and to US and Chinese pig MRV3.

## Main text

The *Reoviridae* family consists of two subfamilies: *Spinareovirinae* and *Sedoreovirinae*, including 9 and 6 genera, respectively. These are icosahedric non-enveloped viruses with a segmented genome of 10 to 12 double-stranded RNA (dsRNA) segments [1]. Viruses belonging to this highly diverse family infect a variety of host species including mammals, reptiles, fish, birds, protozoa, fungi, plants, and insects.

The species Mammalian orthoreovirus (MRV) has been divided into three serotypes according to the capacity of type-specific antisera to neutralize virus infectivity and inhibit hemagglutination, with the prototype isolates being type 1 Lang (T1L), type 2 Jones (T2J), type 3 Dearing (T3D) and Abney (T3A). Recently, this classification has been confirmed through the molecular analyses of the S1 gene encoding for the σ1 protein, which is involved in virus attachment [1]. Moreover, a putative type 4 Ndelle (T4N) has been also proposed [2].

MRVs have long been considered non-pathogenic, although mild respiratory and enteric diseases have occasionally been reported in young animals and children. Several evidences have recently shown that MRVs can cause severe diseases. Cases of neonatal diarrhea and neurological symptoms in children were associated both with MRV2 and MRV3 in Europe and North America [3–5]. These findings highlight the zoonotic potential of MRVs, though the mechanisms of their pathogenicity are not fully understood [5].

Additionally, MRV3 has been recently isolated from piglets with severe diarrhea and respiratory symptoms in China, Korea and the US, also in association with coronaviruses of Porcine Epidemic Diarrhea (PEDV) and Transmissible Gastroenteritis (TGEV), and with Porcine A-C rotaviruses (GARVs, GCRVs) [6–8]. In particular, MRV3 was proven to be pathogenic to pigs [7]. We here report the first isolation and characterization of MRV3 from swine fecal samples in Europe.

In 2015 an important epidemic wave of PED with multiple outbreaks occurred in Italy [9]. Over 200 cases were registered, mainly in high-density pig farm area (Po Valley). The disease was characterized by high morbidity and variable levels of mortality in suckling pigs showing diarrhea and enteritis. These cases were similar to those detected in other European countries, all caused by S-INDEL strains very closely related to each other and to the US Ohio851 strain [9, 10]. A first attempt to isolate PEDV was conducted collecting eleven swine fecal samples at the beginning of the epidemic, between February and March 2015. VERO C1008 cells (ATCC® CRL-1586) were employed according to a previously published method [11]. CPE was detected after the first cell passage in one sample. The supernatant from cell culture showing cytopathic effect (CPE) was submitted to negative staining Transmission Electron microscopy (nsTEM). The nsTEM examination of CPE positive cell culture revealed icosahedral, non-enveloped virus particles with morphological characteristics referable to Reoviridae. RNA was extracted from cell culture and fecal samples using the Nucleospin RNA II kit (Macherey-Nagel, Germany), and analyzed for the presence of MRV using RT-PCR targeting the L1 and S1 fragments, slightly modified from Lelli et al, 2013 [12]. Fecal samples were analyzed by RT-PCR which evidenced the presence of MRV in one fecal sample and in the respective isolate.

Full genome sequencing was conducted using an Illumina MiSeq platform from the isolated virus. Briefly, 100 μl of cell culture supernatants were treated with 250 units of Omnicleave endonuclease (Epicentre, Tebu-bio, Milan, Italy) at 37 °C for 2 h. Viral RNA was extracted from treated supernatants using One for all Vet kit (QIAGEN, Milan, Italy). Sequencing libraries were prepared using TruSeq RNA sample preparation kit v2 (Illumina Inc. San Diego, CA, USA) and sequencing was performed on a Miseq Instrument with MiSeq Reagent Nano Kit v2, 300 cycles (Illumina Inc. San Diego, CA, USA). Sequencing reads were de-novo assembled by Seqman NGen DNASTAR application (version 11.2.1) (DNASTAR, Madison, WI, USA). Genome sequences were available into GenBank under accession numbers KX343200-KX343209.

The phylogenetic trees were constructed with the maximum likelihood method within the MEGA 6.0 software with bootstrap analyses involving 1000 replicates [13]. The best-fit model of the nucleotide substitution was determined using the jModelTest v.0.1.1. The preferred model was the GTR + G model. The topologies were verified with the neighbor-joining method and the Kimura two-parameter model using MEGA 6.0.

The complete genome of the isolate (MRV3/Swine/Italy/224660-4/2015) included segments L1 to L3, M1 to M3 and S1 to S4; each segment showed the highest nucleotide similarity to the sequences reported in Table 1.

**Table 1:** Highest nucleotide identities for each gene segment of the novel MRV3/Swine/Italy/224660-4/2015

Swine-MRV3 Italy 2015	% similarity	MRV strain	Serotype	Lineage	Host	Country	GenBank Acession No.
L1	91.9	T3D	3	II	Human	USA	HM159613
L2	98.9	T3/Pip. kuhlii/Italy/5515-2/2012	3	III	Bat	Italy	KU194659
	98.7	T3/bat/Germany/342/08	3	III	Bat	Germany	JQ412756
	98.7	SI-MRV01	3	III	Human	Slovenia	KF154725
L3	93.7	Abney	3	II	Human	USA	GU589579
M1	91.8	MRV2 Tou5	2	-	Human	France	GU196309
	89.6	SHR-A	3	IV	Pig	China	JX415468
M2	91.0	BatMRV1-IT2011	1	-	Bat	Italy	KT900699
M3	91.4	BM-100	3	III	Pig	USA	KM820749
	90.5	GD-1	3	IV	Pig	China	JX486062
S1	98.4	SI-MRV01	3	III	Human	Slovenia	KF154730
	98.2	T3/Pip. kuhlii/Italy/5515-2/2012	3	III	Bat	Italy	JQ979272
	97.7	T3/bat/Germany/342/08	3	III	Bat	Germany	JQ412761
S2	93.7	T1 Lang	1	-	Human	USA	L19774
	92.4	T3 Abney	3	II	Human	USA	GU589584
S3	93.9	T3/bovine/Maryland /cl. 31/1959	3	III	Bovine	USA	U35357
	92.4	FS03	3	III	Pig	USA	KM820762
S4	94.5	SHR-A	3	IV	Pig	China	JX415473

Note: *L* large segments, *M* medium segments, *S* small segments

**Fig. 1:**
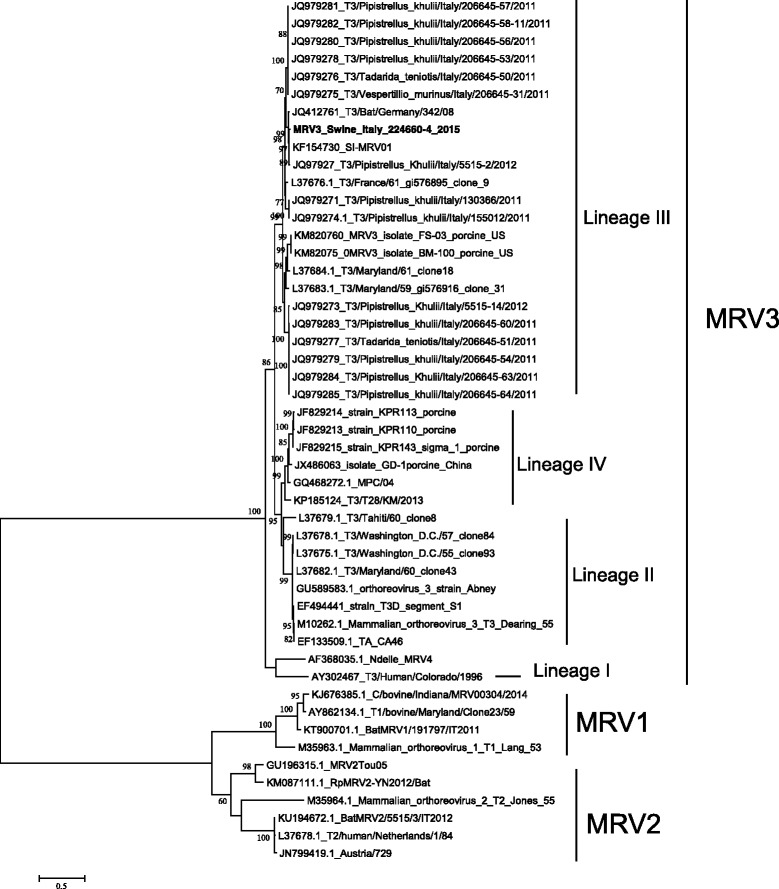
Phylogenetic tree of the complete S1 segment of the Italian MRV3 strain. The tree was performed using the maximum likelihood method, GTR-G model within the IQ-tree software with a bootstrap of 1000 replicates. The Italian strain is underlined

**Fig. 2:**
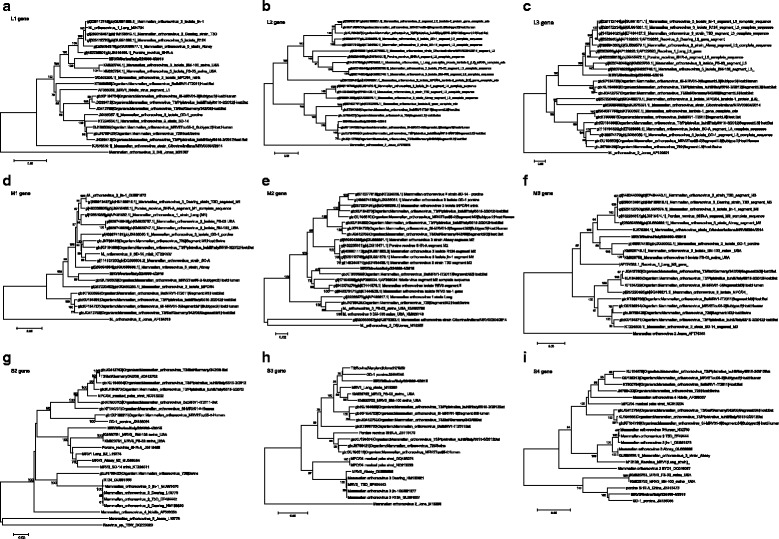
Phylogenetic analysis of L, M and S segments of Italian MRV3 strain. Unrooted neighbour-johining trees based on complete L1, L2, L3, M1, M2, M3, S2, S3, and S4 nucleotide sequences were constructed with 1000 bootstrap replicates in MEGA 6.0. **a** L1 segment; **b** L2 segment; **c** L3 segment; **d** M1 segment; **e** M2 segment; **f** M3 segment; **g** S2 segment; **h** S3 segment; **i** S4 segment. Bootstrap values higher than 60 % was shown. Italian MRV3 strain was reported in bold face

Based on S1 phylogeny, the novel swine MRV strain belonged to the lineage III of the MRV3 and was closely related to human and bat strains and two US porcine MRV3s recently described as associated to PED outbreaks [7] (Fig. 1). It shares the highest nucleotide identity with a human MRV3 Sl-MRV01 (98.4 %) detected from a child with acute gastroenteritis in Slovenia [4] and with an Italian MRV3 bat isolate T3/Pipistrellus Kuhlii/Italy/5515-2/2012 (98.2 %) [12]. The other segments L1, L3, M1, M3, S2, S3 and S4 of the Italian strain were related to US porcine MRV3 (Fig. 2a, c, d, f, g, h and i); In particular S2 and S3 phylogeny indicated monophyletic groups with US and Chinese pig MRV3 strains and human T1L whereas the S4 phylogeny revealed a separated group formed by Italian, US and Chinese pig MRV3 strains. Interestingly, the other two segments L2 and M2 were closely related to MRVs of bat origin, belonging to serotype 3 and 1 respectively [12, 14] (Fig. 2b and e).

In this study, we describe the finding of a MRV3 associated with a PED outbreak in Italy. A similar association was reported in the US during the 2013-2015 PED epidemic, with mortality up to 100 % in affected farms [7]. Porcine MRV3s, placed in lineage IV and frequently associated with other enteric viruses [6], were also described in pigs suffering diarrhea in South Korea. Interestingly, the Italian and US porcine MRV3 associated to PED outbreaks were characterized by a S1 gene highly related to European bat strains and both fall into lineage III, differently from the South East Asian MRV3 porcine isolates. The study of potential synergic effects between PEDV and MRV3 is crucial, considering the PED impact on the swine industry.

Based on the L2 and S1 genetic distances, it appears that the swine and bat Italian MRV3 are highly correlated. Such evidence arises questions on the epidemiological link between pigs and Kuhl’s pipistrelle common in anthropized and urban environments. However, the absence of data on the MRVs distribution and genetic characteristics in Europe prevents any hypothesis on the most likely epidemiological links between bats, pigs and humans. The distribution of MRV3 among pigs and bats could probably be widespread in Europe, although it still needs to be further investigated. Pigs harbor a variety of viruses in their gastro-intestinal tract; not all of them cause diseases but many are related to human viruses, including Noroviruses, Rotaviruses and Astroviruses [15]. The findings reported herein highlight the arising potential role of pigs as a reservoir and amplification host of emerging zoonotic viruses.

## Abbreviations

dsRNA, double strand RNA; GARVs, GCRVs, porcine A-C rotaviruses; MRV3, mammalian orthoreovirus type 3; nsTEM, negative staining transmission electron microscopy; PEDV, porcine epidemic diarrhea; T1L, type 1 Lang; T2J, type 2 Jones; T3A, type 3 Abney; T3D, type 3 Dearing; T4N, type 4 Ndelle; TGEV, transmissible gastroenteritis
